# Coding-Complete Sequence of *Cowpea Mild Mottle Virus* Isolated from Iraq

**DOI:** 10.1128/mra.01224-22

**Published:** 2023-02-01

**Authors:** Ahmed Kareem, Osamah Alisawi, Fadhl Al Fadhl

**Affiliations:** a Plant Protection Department, Faculty of Agriculture, University of Kufa, Najaf, Iraq; DOE Joint Genome Institute

## Abstract

Here, we report the coding-complete sequence of Cowpea mild mottle virus (CPMMV; strain Hilla) in cowpea using total transcriptome sequencing (RNA-seq) and bioinformatics analyses. The sequence length was 8,119 bp long and has 6 open reading frames. The genome was phylogenetically close to two isolates collected from Ghana.

## ANNOUNCEMENT

**FIG 1:**
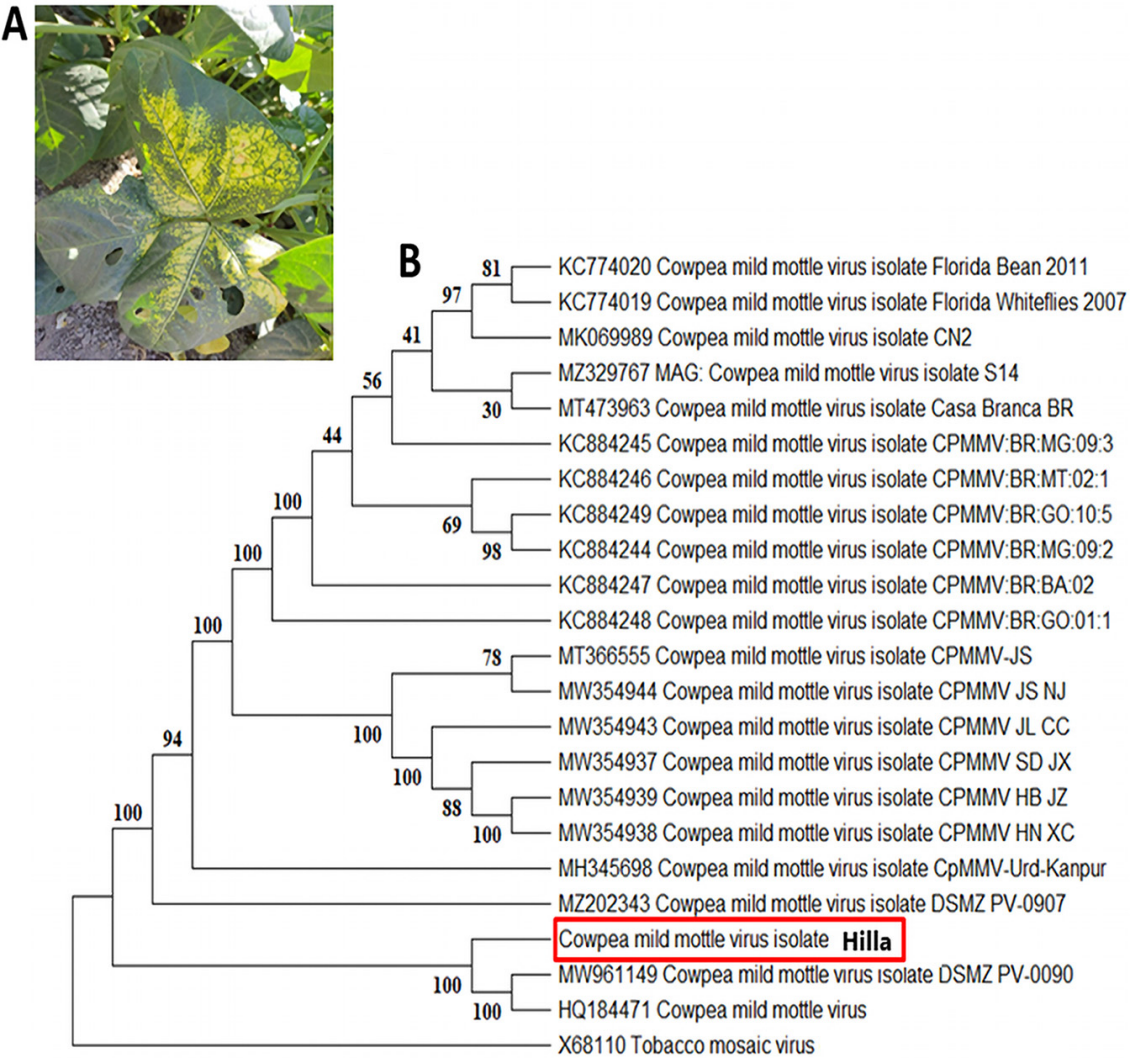
Leaves of cowpea showed yellowing blotches (A). The tree was built by Molecular Evolutionary Genetics Analysis-11 (MEGA-11). The alignment of 23 full-genome nucleotide sequences was performed with ClustalW. A maximum likelihood phylogenetic tree of full-length sequences was constructed using the best substitution model Hasegawa-Kishino-Yano (HKY), and the tree was inferred with 500 bootstraps. The phylogeny showed that Hilla and two strains from Ghana (MW961149 and HQ184471) were closely related (B).

*Cowpea mild mottle virus* (CPMMV; family *Betaflexiviridae*), a member of the *Carlavirus* genus transmitted by whiteflies, first occurred epidemically in Ghana on cowpea in 1973, and the virus has since spread across the globe. In common beans, CPMMV is reemerging after first appearing in soybeans. While it primarily infects Fabaceae, it is also capable of infecting hosts from Solanaceae and Lamiaceae (1). The CPMMV genome is composed of single-stranded, positive-sense RNA (+ssRNA) that is polyadenylated at the 3′ end and capped at the 5′ end. There are a total of six open reading frames (ORFs) in the genome of CPMMV (2). A previous PCR-based study confirmed the existence of the virus and showed a high diversity of CPMMV sequences in Iraq (3). In April 2021, most of the cowpea fields in middle Iraq showed CPMMV-like symptoms, resulting in severe systemic chlorosis and necrosis on the leaves and malformations (Fig. 1A). The infection rate was determined by the formula (number of infected plants/total number of plants in the field) × 100 (4) and ranged between 1 and 21.4%. Leaves showing typical symptoms were collected from pathogenic cowpea plants in the Hilla region, Babylon Province, on 3 November 2021, then cut into a square with a size of 0.5 by 0.5 cm, immersed in a 5× volume of RNAlater in an Eppendorf tube, and sent to the DNA Link company in the Republic of Korea for sequencing. RNA was extracted from the plant sample according to the manufacturer’s instructions using the RNeasy plant mini kit (Qiagen, Hilden, Germany). The sequencing library was prepared with the TruSeq total RNA library prep kit and subjected to whole-genome sequencing (platform, Novaseq 6000; application, whole-transcriptome sequencing [WTS]/mRNA), and the reads were trimmed by the Trimmomatic-0.39 program (5). The corresponding RNA-seq library consists of 53,728,592 clean and paired-end reads of 101 bases in length. A reference genome database was constructed using 5040 virus sequences downloaded recently from NCBI GenBank. Geneious Prime 2022.2.2 software (6) was used to map the clean and paired-end reads against the reference genome database. The RNA-seq data were mapped against the reference genome database using Geneious RNA mapper (medium-low sensitivity). This mapping analysis showed a high number of reads mapped to *Cowpea mild mottle virus* isolate DSMZ PV-0090 (GenBank accession number MW961149). To produce a consensus sequence of 8,119 bp and count the actual number of mapped reads, mapping against the isolate DSMZ PV-0090 was applied specifically, and 11,300,890 reads were assembled. The sequence has a GC content of 41.9% and an average coverage depth of 637,895. The consensus sequence was extracted and aligned with the reference genome and then annotated in Geneious using the Open Reading Frame Finder and the basic local alignment search tool (BLASTx) to predict protein domains. All tools were run with default parameters unless otherwise specified. It encodes six open reading frames that represent replicase, three proteins of gene block (TGBp1, TGBp2, and TGBp3), coat protein (CP), and nucleic acid-binding protein. The coding-complete sequence of the virus was deposited in GenBank under accession number ON993892, and the strain was named Hilla. The phylogeny showed a close relationship between Hilla and two strains from Ghana (MW961149 and HQ184471), indicating a common ancestor of the isolates (Fig. 1B). The diagnosis of the coding-complete sequence of the CPMMV virus is essential to identify the emergence and spread factors of such viruses that threaten legume fields in Iraq.

### Data availability.

This whole-genome shotgun project has been deposited in GenBank under the accession number SRR22382642. The version described in this paper is the first version. The coding-complete sequence of CPMMV has been deposited in GenBank under accession number ON993892.
